# Mesenchymal Stromal Cells as Potential Antimicrobial for Veterinary Use—A Comprehensive Review

**DOI:** 10.3389/fmicb.2020.606404

**Published:** 2020-12-01

**Authors:** Keith A. Russell, Livia C. Garbin, Jonathan M. Wong, Thomas G. Koch

**Affiliations:** ^1^Department of Biomedical Sciences, Ontario Veterinary College, University of Guelph, Guelph, ON, Canada; ^2^Clinical Veterinary Sciences Department, School of Veterinary Medicine, Faculty of Medical Sciences, The University of the West Indies, St. Augustine, West Indies

**Keywords:** veterinary medicine, antimicrobial resistance (AMR), animal models, mesenchymal stem (stromal) cell, cellular therapy

## Abstract

The emergence of “superbugs” resistant to antimicrobial medications threatens populations both veterinary and human. The current crisis has come about from the widespread use of the limited number of antimicrobials available in the treatment of livestock, companion animal, and human patients. A different approach must be sought to find alternatives to or enhancements of present conventional antimicrobials. Mesenchymal stromal cells (MSC) have antimicrobial properties that may help solve this problem. In the first part of the review, we explore the various mechanisms at work across species that help explain how MSCs influence microbial survival. We then discuss the findings of recent equine, canine, and bovine studies examining MSC antimicrobial properties in which MSCs are found to have significant effects on a variety of bacterial species either alone or in combination with antibiotics. Finally, information on the influence that various antimicrobials may have on MSC function is reviewed. MSCs exert their effect directly through the secretion of various bioactive factors or indirectly through the recruitment and activation of host immune cells. MSCs may soon become a valuable tool for veterinarians treating antimicrobial resistant infections. However, a great deal of work remains for the development of optimal MSC production conditions and testing for efficacy on different indications and species.

## 1. Introduction

Antimicrobial resistance (AMR) is a growing concern in all clinical populations, with few treatment options for those afflicted. Resistance is caused by unnecessary or superfluous antimicrobial use or even misuse associated with suboptimal dosage or duration (Guardabassi et al., 2018). In the last few years, the American Veterinary Medical Association (AVMA), Federation of Veterinarians of Europe (FVE), and Canadian Veterinary Medical Association (CVMA) have released a joint statement on responsible and judicious use of antimicrobials and have published guidelines for appropriate veterinary antimicrobial use^1^. Further, many federal agencies around the world are moving to reduce overall use of antimicrobials in animals. For example, Canada has restricted the sale of medically important antimicrobials for veterinary use by changing their status to prescription drugs^2^. Clinicians may want to consider alternative or complementary strategies in order to treat microbial infections.

Mesenchymal stromal cells (MSCs) have long been explored in regenerative medicine as raw material for engineering tissues or as immunomodulatory agents for treatment of inflammatory diseases (Devireddy et al., 2017). More recently, MSCs have shown promise as a potential treatment to address AMR. MSCs have antimicrobial properties whose mechanisms are still being uncovered. They are known to both secrete antimicrobial molecules that directly interact with pathogens as well as other factors that boost the antimicrobial activity of host immune cells (Maxson et al., 2012; Alcayaga-Miranda et al., 2017). In proof of concept studies, MSCs have shown strong synergy with existing antibiotic treatments to penetrate biofilm infections (Johnson et al., 2017) as well as capacity to serve as antifungal (Yang et al., 2013; Arango et al., 2018), antiviral (Kang et al., 2005; Kniazev et al., 2012; Khatri et al., 2018), and antiparasitic (Spekker et al., 2013) agents.

Over-prescription of broad-spectrum antibiotics in both human and veterinary medicine are creating a growing need for novel methods of disease treatment (Shallcross and Davies, 2014; Martin et al., 2015). In 2015, the WHO recognized the growing threat of AMR and recommended a global action plan built around the interdisciplinary “One Health” approach to combat AMR at the Animal-Human-Ecosystems interface (World Health Organization, 2017). Recommendations include an overall reduction of medically-important antimicrobials in the treatment of food-producing animals and the complete restriction of such antimicrobials for reasons of growth promotion or prophylactic use. If MSC antimicrobial properties can translate into viable treatment options, they could supplant much of the antimicrobials currently in use in animals. In this review, we will explore the veterinary literature regarding MSCs as antimicrobials. Although this is an emerging field of study, we will outline possible mechanisms and examine potential synergism to be found in combination therapies as well as possible deleterious effects of such an approach.

## 2. Antimicrobial Effect of MSCs

MSCs have demonstrated antimicrobial effects both *in vitro* and *in vivo* with many different mechanisms implicated throughout the literature. Elucidating mechanisms can be challenging due to the fact that mechanisms can vary across donor species as well as target species of bacteria (Meisel et al., 2014; Mezey and Nemeth, 2015; Maria Holban et al., 2016; Rodríguez-Milla et al., 2020). Other variables can further impact the MSC phenotype and frustrate efforts to find consistency across studies such as MSC tissue source and the use of preconditioning protocols (Mezey and Nemeth, 2015; Cortés-Araya et al., 2018; Taguchi et al., 2019). Preconditioning, also known as activating or priming, MSCs through various culture conditions is commonly used to modulate or enhance desirable MSC properties. A number of preconditioning methods have been employed over the years including hypoxia, serum deprivation, and exposure to antagonistic substances to improve MSCs' ability to differentiate or modulate immune cells. Conditions promoting antimicrobial activity are being examined with exposure to cytokines, target bacteria, bacterial components, vitamins, and antibiotics improving both direct and indirect antimicrobial effects (Gupta et al., 2012; Guerra et al., 2017; Johnson et al., 2017; Cahuascanco et al., 2019; Yagi et al., 2020).

### 2.1. Direct Mechanisms of Antimicrobial Effects of MSC

**Figure 1:**
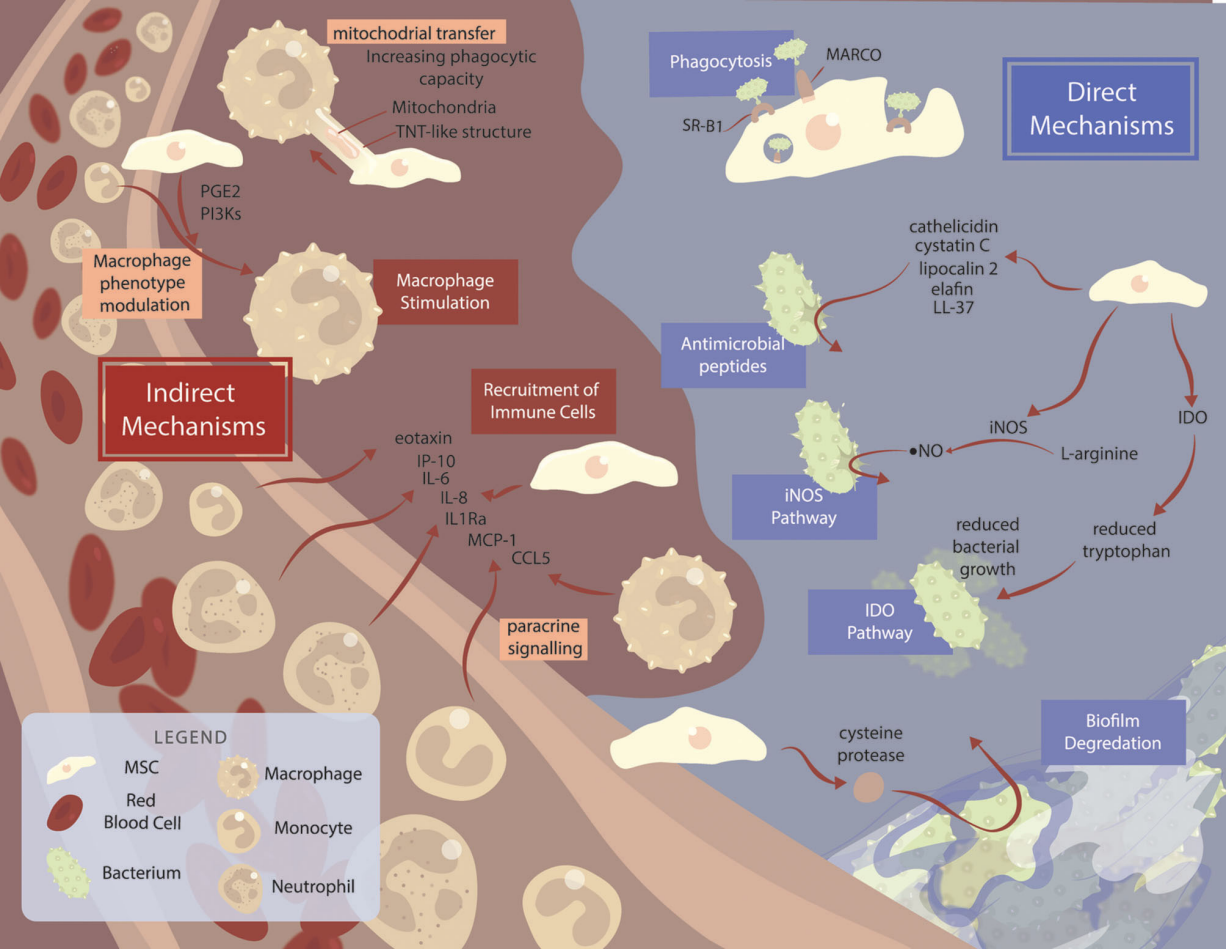
Mechanisms of mesenchymal stromal cell-mediated killing across species. Summary of the major mechanisms and pathways used by MSCs. MSCs are often able to exhibit antimicrobial properties without stimulation, although various factors can improve MSC functionality. Direct mechanisms of MSC-mediated bacterial killing (blue background) include MARCO and SR-B1 receptor-mediated phagocytosis, antimicrobial peptide production, and IDO and iNOS pathways. Degradation of bacterial biofilms via cysteine protease secretion acts as a method of improving antibiotic function in combination therapy. Indirect mechanisms (brown background) include MSC bacterial killing via immune cell recruitment, and macrophage stimulation.

Antimicrobial peptides (AMPs) are key components to MSC antimicrobial efficacy (Figure 1, Table 1). AMPs are short strings of amino acids co-expressed in clusters as a natural defense to bacteria, yeasts, fungi, and cancer cells (Vizioli and Salzet, 2002; Neshani et al., 2019). Over 1,200 known AMPs exist and are produced by organisms ranging from prokaryotes to higher animals (Lai and Gallo, 2009). AMPs primarily facilitate microbial killing through disruption of the microbial cell membrane. In addition, AMPs also modulate host innate immune cells mounting an orchestrated defense to microbes (Lai and Gallo, 2009). Several AMPs have been identified to be secreted by MSCs contributing to their overall antimicrobial function, with species-dependent expression of specific AMPs. Among the more broadly studied families of AMPs are cathelicidin, defensin, and lipocalin.

**Table 1:** Mechanisms of MSC antimicrobial effects.

**MSC origin**	**Study type (model)**	**MSC pre-activation**	**Bacteria**	**Mechanism**	**Outcome**	**Reference**
**Direct mechanisms**						
Equine PB	*In vitro*	No	*E. coli* and *S. aureus*	Cystatin C, elafin, lipocalin 2, cathelicidin secretion	MSC and MSC conditioned media inhibited bacterial growth.	Harman et al., 2017
Human CB	*In vitro*/*in vivo* (mouse)	Yes	*E. coli*	β-defensin secretion	*In vitro*: growth of bacteria was significantly inhibited by MSCs or their conditioned medium. siRNA mediated knockdown of TLR-4 abolished antibacterial effects of MSCs. *In vivo*: intratracheal transplantation of MSCs reduced alveolar congestion, hemorrhage, neutrophil infiltration, and wall thickening 1 day post-*E. coli* intratracheal inoculation.	Sung et al., 2016
Human BM	*In vitro*/*in vivo* (mouse)	No	*E. coli, P. aeruginosa, S. aureus*	LL-37 secretion	*In vitro*: MSC and MSC conditioned media decreased bacterial growth in comparison to controls. *In vivo*: MSCs reduced bacterial growth in lung homogenates and bronchoalveolar lavage fluid.	Krasnodembskaya et al., 2010
Murine AT	*In vivo*	Yes	*S. aureus*	Cathelicidin secretion	Administration of antibiotics or MSC alone did not significantly reduce bacterial burden at wound site. TLR3 ligand-activated MSC with antibiotic therapy was the only treatment that significantly reduced bacterial burden at wound site.	Johnson et al., 2017
Murine BM	*In vitro*	No	*M. smegmatis, M. bovis*	Cathelicidin secretion	MSCs induced killing of *M. smegmatis* and *M.bovis* but were unable to kill *M. tuberculosis*.	Naik et al., 2017
Equine PB	*In vitro*	No	*P. aeruginosa, A. viridans, A. baumannii, S. epidermis, S. aureus*, MRSA	Cysteine protease secretion	MSC conditioned media inhibited bacterial growth for all bacteria tested. Cysteine protease secretion was found to inhibit biofilm formation as well as improve efficacy of antibiotics against mature biofilms of MRSA.	Marx et al., 2020
Human BM, CB	*In vitro*	No	*M. tuberculosis*	Direct phagocytosis, nitric oxide secretion	Phagocytosed bacteria did not replicate within MSCs, while showing a decline in numbers over 7 days	Khan et al., 2017
Human BM	*In vitro*	Yes	*S. aureus, S. epidermis, E. faecium*, group B streptococci, *T. gondii*, human cytomegalovirus	Indoleamine 2,3-dioxygenase pathway	MSCs exhibited broad-spectrum antimicrobial effector function. Addition of IDO inhibitors or tryptophan restored bacterial growth.	Meisel et al., 2011
Murine BM	*In vitro*	Yes	*S. aureus, S. epidermis, T. gondii*	iNOS pathway	Failed to inhibit *S. aureus* and *S. epidermis*. Intracellular growth of *Toxoplasma gondii* parasites was attenuated, with inhibitory effect being partially blocked by the iNOS-specific inhibitor NGMMA.	Meisel et al., 2011
**Indirect mechanisms**						
Human BM	*In vitro*/*in vivo* (rat)	No	*E. coli*	Macrophage differentiation into M1-like and M2-like macrophages	MSCs enhanced human macrophage phagocytosis of unopsonized bacteria and enhanced bacterial killing when compared with untreated macrophages. PGE2 and PI3K were key mediators of M1 macrophage induction.	Rabani et al., 2018
Human PDL	*In vitro*	Yes	None	RANTES, eotaxin, IP-10, MCP-1, IL-6, IL-8, and IL-1ra	*P. gingivalis* total protein extract pre-treatment induced higher secretion of inflammatory markers and chemokines. Increased recruitment of neutrophil-differentiated human promyelocytic leukemia HL-60 cells was seen with increased production of intracellular reactive oxygen species.	Misawa et al., 2019
Murine AT	*In vivo*	Yes	*S. aureus*	Chemokine CCL2 release	Increased neutrophil phagocytosis, monocyte recruitment, M2 macrophage induction.	Johnson et al., 2017
Human BM	*In vivo* (mouse)	No	*E. coli*	Mitochondrial transfer from MSCs to macrophages	MSC administration was associated with enhanced alveolar macrophage phagocytosis.	Jackson et al., 2016

*E. coli, Escherichia coli; S. aureus, Staphylococcus aureus; MSC, mesenchymal stromal cell; AT, adipose tissue; BM, bone marrow; CB, cord blood; EM, endometrium; PB, peripheral blood; PDL, periodontal ligament; TLR-4, Toll-like receptor 4; P. aeruginosa, Pseudomonas aeruginosa; TLR-3, Toll-like receptor 3; M. smegmatis, Mycobacterium smegmatis; M. bovis, Mycobacterium bovis; MCP-1, monocyte chemoattractant protein-1; CCL5, chemokine ligand 5; IL-6, interleukin 6; IL-8, interleukin 8; IRPA, imipenem-resistant P. aeruginosa; LPS, lipopolysaccharide; PGE2, prostaglandin E2; PI3K, phosphatidylinositol 3-kinase; RANTES, regulated on activation normal T cell expressed and secreted; IP-10, interferon γ inducible protein 10; P. gingivalis, Porphyromonas gingivalis; IL-1ra, interleukin 1 receptor antagonist; CCL2, chemokine ligand 2; M. tuberculosis, Mycobacterium tuberculosis; S. epidermis, Staphylococcus epidermidis; E. faecium, Enterococcus faecium; A. viridans, Aerococcus viridans; A. baumannii, Acinetobacter baumannii; NGMMA, N-G-monomethyl-L-arginine; MRSA, methicillin-resistant S. aureus; IDO, indoleamine 2,3-doxygenase; PMN, polymorphonuclear neutrophil granulocytes; NET, neutrophil extracellular trap*.

Cathelicidins are a major family of AMPs with prominent roles in innate immunity. Notably, cathelicidins across different species can vary their mechanism of action, albeit bacterial membrane disruption, and cell lysis generally occur (Schneider et al., 2016; Scheenstra et al., 2019). In humans, cathelicidin LL-37 has been implicated in the direct bacterial killing effects of MSCs (Guerra et al., 2017; Ren et al., 2019). LL-37 has bactericidal properties as well as the ability to decrease cytokine and endotoxin levels in septic models. Investigators identified LL-37 activity as crucial to MSC antimicrobial activity *in vitro* as well as in an *in vivo* mouse model using human MSCs (Krasnodembskaya et al., 2010). Yagi et al. (2020) further found LL-37 activity from adipose-derived MSCs was dependent on 1,25-dihydroxy vitamin D3. 1,25-dihydroxy vitamin D3 supplementation enhanced LL-37 production relative to MSCs under standard culture conditions, whereas treatment with a vitamin D receptor inhibitor nullified the antibacterial response. *In vivo* studies using allogenic murine MSCs have also implicated cathelicidin as a key AMP for bacterial killing (Johnson et al., 2017). Cathelicidin acts through TLR2/4-IRAK-4-dependent pathways in order to establish effective killing of mycobacteria (Naik et al., 2017). Interestingly, *M. tuberculosis* (Mtb) has developed a survival mechanism that disrupts this pathway and suppresses the antimicrobial effect of BM-MSCs via downregulation of *CAMP* gene expression (Naik et al., 2017). This suggests that a panel of AMPs might be necessary to overwhelm the defenses of certain microbes.

β-defensins are cysteine-rich cationic proteins with sizes ranging from 18 to 145 amino acids (Kim, 2014). These molecules similarly form pores in bacteria resulting in lysis (Esfandiyari et al., 2019). The presence of β-defensins have not always been detected in human MSC studies. Sutton et al. (2016) found no presence of β-defensin 2 or β-defensin 3 after MSC exposure to *P. aeruginosa, S. aureus*, and *Streptococcus pneumonia*, and attributed all bactericidal effect to LL-37 release. Conversely, Ren et al. (2019) identified human β-defensin 2 in both *P. aeruginosa*-stimulated and unstimulated MSCs. Other investigators identified β-defensin secretion from human MSCs via TLR-4 signaling as the key mechanism of paracrine *in vitro* antibacterial effect after *E. coli* exposure (Sung et al., 2016). Sung et al. also demonstrated similar β-defensin secretion from human MSCs treating *E. coli* in a mouse model. In cows, AMP gene expression of β-defensin 4A (bBD-4A) in addition to NK-lysine 1 (NK1) was found in fetal MSCs, while cathelicidin 2, hepcidin, and IDO expression were not found (Cahuascanco et al., 2019). Co-culture with *S. aureus* increased gene expression of bBD-4A and NK1.

Lipocalin 2 is an AMP that works by sequestering iron-laden siderophores, thus depriving bacteria of iron and limiting bacterial growth (Flo et al., 2004). Higher expression of lipocalin 2 was found in syngeneic murine MSCs after exposure to gram-negative bacterial pneumonia in an *in vivo* mouse model (Gupta et al., 2012). When lipocalin 2 was blocked in this study, the bacterial clearance effect observed with MSCs was lost. Expression of lipocalin 2 could be upregulated through MSC activation with LPS and TNFα. In horses, it was also found that LPS stimulation led to increased lipocalin 2 expression in equine MSCs (Cortés-Araya et al., 2018). In another equine MSC study, lipocalin 2 was also detected along with cathelicidin, cystatin C, and elafin (Harman et al., 2017). While AMPs are significant to MSCs' response to microbial challenge, they are not the only mechanisms at work.

Indoleamine 2,3-dioxygenase (IDO) expression in MSCs has also been involved in response to bacteria in humans. IDO acts to reduce local tryptophan levels thus inducing broad-spectrum antimicrobial activity (Däubener et al., 2009). MSCs stimulated with the inflammatory cytokines TNFα, IL1β, and IFNγ upregulated IDO expression, resulting in a reduction of bacterial growth (Meisel et al., 2011). TNFα and IL1β alone failed to restrict bacterial growth, yet both cytokines upregulated the IFNγ-mediated IDO-activity. Notably, addition of IDO inhibitors or tryptophan restored bacterial growth, confirming IDO as a key mechanism of antimicrobial effect. In contrast, IDO expression by murine MSCs was not found in *in vivo* mouse studies (Meisel et al., 2011). The authors found murine MSCs were unable to inhibit *S. aureus* growth due to the lack of IDO but were effective at inhibiting intracellular growth of *T. gondii* parasites via the inducible nitric oxide synthase mechanism (Meisel et al., 2011). Interestingly, infection with human cytomegalovirus (HCMV) suppressed human MSCs' ability to induce bacterial and parasitic killing, due to HCMV's ability to inhibit the IFN-γ pathway (Meisel et al., 2014). Relevantly, while some researchers have found no expression of IDO in equine MSCs (Carrade et al., 2012), others have found that upregulation of IDO expression can be induced by priming equine MSCs with exposure to the TLR3 agonist poly I:C (Cassano et al., 2018). As antimicrobial effects of IDO in equine cells have not been assessed, future studies are warranted to further explore this mechanism in an equine population.

Another mechanism at play is MSCs' ability to phagocytose Mtb (Khan et al., 2017). Direct internalization of Mtb relies on the macrophage receptor with collagenous structure (MARCO) and SR-B1 receptors. Rapamycin exposure increased lipidation of microtubule-associated light chain-3. Furthermore, no change in viability was seen *in vitro* after 7 days of infection, and internalized Mtb counts decreased over 7 days. They also found nitric oxide (NO) secretion by MSCs which further restricted Mtb growth. Bacterial internalization has also been noted in other studies, where human MSCs were found to internalize *S. aureus* (Josse et al., 2014; Guerra et al., 2017). Guerra et al. noted correlations between production of IL-6 by MSC and bacterial internalization, although mechanistic studies were not performed to clarify this relationship.

Biofilm development is a hallmark of antibiotic resistance. Biofilms are a bacteria-produced polymer matrix, resulting in increased resistance to disinfectants, antibiotics, and immune cells (Wu et al., 2015). Recent evidence has suggested MSCs have potential in breaking down biofilms, which is clinically relevant as infections that reach the biofilm stage have increased antimicrobial tolerance by 100–1,000-fold (Olsen, 2015). As a consequence, reaching effective antibiotic levels *in vivo* becomes unattainable due to the associated side effects and toxicity (Olsen, 2015). MSCs present a strategy to increase efficacy of conventional antibiotics via degradation of the biofilm layer and increased antibiotic penetration. Marx et al. investigated the *in vitro* effects of the MSC conditioned media (CM) against a variety of bacteria. Investigators found inhibition of biofilm formation and growth in *P. aeruginosa, S. aureus*, and *S. epidermidis*, although this was not consistent against all bacterial strains (Marx et al., 2020). In extension to these findings, the presence of cysteine protease was identified in the equine MSC CM, which was found to inhibit MRSA biofilms via reduction of extracellular protein content and allowed for better penetration of conventional antibiotics (Marx et al., 2020). These preliminary findings suggest potential for MSC-antibiotic combination therapy, although further studies must be done to confirm clinical treatment viability.

### 2.2. Indirect Mechanisms of Antimicrobial Effects of MSC

MSCs have further demonstrated the ability to interact with the host immune system via paracrine factors and direct cell-cell interactions. Macrophages are key immunological players, having roles in tissue repair, homeostasis, and bacterial autophagy (Bah and Vergne, 2017; Doster et al., 2018). Furthermore, macrophages can be induced into the anti-inflammatory M2 phenotype or the pro-inflammatory M1 phenotype (Jayasingam et al., 2020). Johnson et al. (2017) identified M2 macrophage induction by activated allogeneic murine MSCs in infected tissues, whereas untreated infected tissues had a M1 dominant macrophage population. Bacterial killing has been attributed to the ability of MSCs to induce the M1 macrophage phenotype. Notably in this study, treatment with non-activated MSCs resulted in a mixed population of M1 and M2 macrophages. M2 macrophages were hypothesized to improve wound healing, which was consistent with the improved physical and histological appearance of the activated MSC treatment group when compared with the other treatment groups (Johnson et al., 2017). Similar findings were shared by Rabani et al. (2018) where non-activated human MSCs were in the same way capable of affecting macrophage phenotype, inducing a mixed population of M2 and M1 macrophages in a rat model. It was found that MSC modulation of human macrophages was dependent on prostaglandin E2 and phosphatidylinositol 3-kinase, which resulted in effective phagocytosis of unopsonised bacteria (Rabani et al., 2018). MSC administration can also result in enhanced alveolar macrophage phagocytosis as shown in a recent mouse study (Jackson et al., 2016). A tunneling nanotube (TNT)-like structure was used to transfer mitochondria from human MSCs to macrophages both *in vitro* and *in vivo*, which resulted in improved macrophage phagocytic capacity and bioenergetics (Jackson et al., 2016). Direct MSC-macrophage cell contact was found to optimize mitochondrial transfer, although blockage of MSC TNT formation via cytochalasin B did not fully abrogate mitochondrial transfer due to exosome-mediated mitochondrial transfer. *In vivo* studies found TNT formation was required for antimicrobial efficacy of MSCs, implying cell contact-dependent transfer is key for macrophage polarization. Similar results were seen in a rodent model using human MSCs, where MSCs enhanced macrophage phagocytosis of *E. coli*, and further enhancements in phagocytic activity were seen with addition of endotoxin and TNFα (Devaney et al., 2015). Lee et al. proposed another mechanism for macrophage stimulation. Allogeneic human MSCs were found to release keratinocyte growth factor (KGF) onto KGF receptors on human monocytes, resulting in enhanced bacterial clearance and decreased apoptosis of monocytes in an *ex vivo* lung model (Lee et al., 2013b). Other studies identified high levels of CCL2 released by MSCs *in vitro*, which is known to induce recruitment of inflammatory monocytes (Johnson et al., 2017). Higher levels of CCL2 were released by poly I:C-activated MSCs compared to non-activated MSCs.

Neutrophils have demonstrated similar phagocytic enhancements seen in macrophages. MSCs were found to enhance polymorphonuclear neutrophil granulocyte (PMN) bacterial uptake via secretion of IL-6, IL-8, and MIF cytokines (Brandau et al., 2014). These molecules bind to receptors CXCR1 and CXCR2 on neutrophils to mediate PMN recruitment and activation (Lazennec and Richmond, 2010; McDonald and Kubes, 2011). Neutrophils are further known to produce neutrophil extracellular traps (NET) which aid in preventing spread of bacteria and mediate killing (Hirschfeld, 2014). Chow et al. identified increased NET area produced per cell after incubation with CM from poly I:C-activated human MSCs when compared to non-activated MSC or control neutrophils in a mouse model (Chow et al., 2020), albeit NET formation was seen in all groups. Neutrophil phagocytosis was also seen in this study, with a similar activation-augmented effect.

Furthermore, activation of MSCs has been shown to enhance other immune regulatory properties. Human periodontal ligament-derived MSCs (PDLSC) stimulated with *P. gingivalis* total protein extract (PgPE) secreted inflammatory markers and chemokines including RANTES, eotaxin, IFNγ, inducible protein 10 (IP-10), IL-6, IL-8, and interleukin receptor antagonist protein (IL-1ra) (Misawa et al., 2019). The authors concluded PDLSCs were key in recruiting immune cells to infected tissues, with unstimulated MSCs having negligible levels of chemokines. MSC exposure to *S. typhimurium* and *L. acidophilus* has also resulted in higher transcription of immunomodulatory genes COX2, IL-6, and IL-8 as well as increased PGE2 secretion (Kol et al., 2014). These findings are supported in equine populations where in addition to AMP expression, investigators identified upregulated expression of immunomodulatory genes MCP-1, IL-6, IL-8, and CCL5 in equine MSCs after bacterial challenge, thus pointing to immune cell recruitment and activation as mechanisms of microbial killing (Cortés-Araya et al., 2018). MSCs have further been found to increase immunomodulatory activity after minocycline exposure. Minocycline induced phosphorylation of transcriptional nuclear factor-kB (NFkB) in human MSCs, resulting in decreased LL-37 production, increased IL-6 production, and overall net reductions in *S. aureus* bacterial load (Guerra et al., 2017). While it is surprising that lower LL-37 production would lead to reduced bacterial survival, it shows that combining antibiotics with MSCs must be examined closely for both synergistic and antagonistic effects as will be discussed below.

### 2.3. Examples of Antimicrobial Effect of MSC in Domestic Animals and Models

When looking at an overview of the MSC antibacterial research undertaken in the veterinary field, it is useful to look first at the broad strokes of the different approaches to this problem that research groups have taken. A good place to start is to examine what source of MSCs were used. It could be argued that all of the studies to be discussed here were guided by the clinical practicality of using allogeneic cells or cell-free preparations over autologous cells as they have been repeatedly shown to be well-tolerated (Zhang et al., 2015; Bogatcheva and Coleman, 2019). If the aim is to supplement or replace antibiotics, then an off-the-shelf cellular product is preferred. When looking at the broader field of MSC study historically, the most common source tissues from which MSCs are isolated are BM and adipose tissue (AT) (Xu et al., 2017). It should come as no surprise that these sources were also most common in this subset of studies. Ease of isolation was likely a factor in choosing BM or AT for some (Johnson et al., 2017; Cortés-Araya et al., 2018; Cahuascanco et al., 2019; Bujňáková et al., 2020; Peralta et al., 2020) and peripheral blood (PB) for others (Harman et al., 2017; Marx et al., 2020). Still, potency and lack of immunogenicity were also cited as reasons for choosing certain tissue sources, which led more than one group to isolate MSCs from fetal sources of BM, AT, or the amniotic membrane (Cahuascanco et al., 2019; Lange-Consiglio et al., 2019; Peralta et al., 2020). Although fetal and perinatal tissue-derived MSCs have been reported to be superior to their adult-derived counterparts in their proliferative potential and hypoimmunogenicity (Deus et al., 2020), it is as yet unclear what effect, if any, their more primitive state might have on their antimicrobial activity.

Another trend in the field of MSC study that can be seen reflected among these studies is the move toward cell-free therapies using the secretome and CM of the cells. MSCs are known to secrete a wide range of potentially therapeutic biomolecules and factors into the extracellular space including growth factors, cytokines, chemokines, extracellular vesicles, and the aforementioned AMPs. The specific makeup of the secretome can vary depending on tissue source and in response to environmental conditions (Al Naem et al., 2020). Consequently, the secretome can be influenced by culture conditions that activate or precondition MSCs to respond to the clinical problem at hand as discussed above. In the articles reviewed here, only two of the studies took strictly a cellular approach (Johnson et al., 2017; Peralta et al., 2020), one assessed both cells and CM (Harman et al., 2017), while the rest worked only with CM (Cortés-Araya et al., 2018; Cahuascanco et al., 2019; Lange-Consiglio et al., 2019; Bujňáková et al., 2020; Marx et al., 2020). Co-culturing equine PB-MSCs with either *E. coli* or *S. aureus*, Harman et al. compared direct contact with transwell separation and found that both the cells and the paracrine factors alone had an inhibitory effect albeit somewhat muted in the case of the transwell setup (Harman et al., 2017). Follow-up experiments with CM from unstimulated MSCs indicated that at least some of the antibacterial factors were secreted constitutively and were not as a result of direct bacterial stimulation. Using cell-free preparations has advantages over using the cells themselves. It removes some safety risks associated with live cell transplantations including immune compatibility and tumorigenicity (Vizoso et al., 2017). CM can also be concentrated for higher potency or lyophilized for cheaper and easier shipment and storage (Bogatcheva and Coleman, 2019). Harman investigated the effects of lyophilization/reconstitution as well as heat inactivation, proteinase K treatment, and freezing/thawing on their equine PB-MSC CM and found that its antimicrobial potency remained regardless of processing method employed (Harman et al., 2017). Cahuascanco et al. (2019) evaluated the effects of concentrating (4x) CM or using CM from activated fetal bovine AT- and BM-MSCs. While the concentrated CM was not directly compared to the non-concentrated CM, the activated CM significantly reduced *S. aureus* proliferation over the 3 h tested both when concentrated and not. MSCs were activated with pre-exposure to *S. aureus*. Activation or preconditioning of MSCs is not unique to antimicrobial studies. It is a strategy that has been used to enhance specific MSC properties including *in vivo* survival and immunomodulation (Lee and Kang, 2020). For example, to bolster immunomodulatory activity, previous studies have employed the TLR3 and TLR4 receptor agonists LPS (Yan et al., 2014; Liu et al., 2016; Kink et al., 2019) and poly I:C (Rashedi et al., 2016; Qiu et al., 2017; Kim et al., 2018). These immune receptor agonists were used similarly to prime canine (Johnson et al., 2017) and equine (Cortés-Araya et al., 2018) MSCs to boost antimicrobial activity as well.

Breaking it down by species, the antimicrobial properties of equine MSCs have been evaluated in recent years although no *in vivo* work has been undertaken to date. As mentioned briefly above, Harman et al. (2017) looked at the effects of PB-MSCs and CM on both *E. coli* and *S. aureus*. Equine dermal fibroblasts were used as control cells as they are known to secrete antimicrobial peptides. In both direct contact and transwell coculture experiments, the MSCs were shown to have an inhibitory effect on both bacterial species equal to or greater than the fibroblasts. They further investigated what secreted AMPs could be responsible for these effects and found cystatin C, elafin, lipocalin 2, and cathelicidin to be expressed at higher levels than fibroblast controls but not β-defensin 1 found in other species. Some of these findings were partially confirmed in a similar equine study that found expression of lipocalin 2, but not β-defensin 1 in equine MSCs derived from bone marrow, endometrium, and adipose tissue (Cortés-Araya et al., 2018). These four AMPs alone could only account for part of the effect as there was still considerable effect after blocking AMP activity (Harman et al., 2017). According to the authors, although the effect of the AMPs are not immediately bactericidal, they depolarize bacterial cell membranes and may serve to increase the efficacy of conventional antibiotics. The authors further identified differential mechanisms used by MSCs to target different bacterial species, where factors >10 kDa inhibited growth of *E. coli* and factors >30 kDa inhibited growth of *S. aureus*. Some caution must be observed when drawing conclusions based on this study alone since penicillin/streptomycin and gentamicin were added to the cell media. Use of these antibiotics certainly may have influenced the results to some extent, although the fractionation experiments clearly show other factors at work (Harman et al., 2017). To build on these findings and correcting for the earlier design flaw, this research group turned more recently to the greater challenges of potentially treating biofilms and multidrug resistant (MDR) bacteria (Marx et al., 2020). Antibiotic-free CM from both equine PB-MSCs and dermal fibroblasts were measured with DMEM or DMEM with antibiotics as controls. In the initial experiments, four bacterial species commonly associated with skin wounds as well as *S aureus* were tested in three different states: planktonic and developing or established biofilms. For planktonic bacterial cocultures, there was significantly better inhibition of bacterial growth in the antibiotic treatment than MSC CM with which only some inhibition was seen in 4/5 of the species. Separately, MSC CM was shown to diminish developing and mature biofilms in most cases often as effectively as antibiotics. However, when looking at the biofilms of methicillin-resistant *S. aureus* (MRSA), only MSC CM was capable of significant growth inhibition. This growth reduction was attributed in part to cysteine protease activity of cathepsins and others seen highly expressed in a protease array. This mechanism was validated by the use of a protease inhibitor, which reduced the MSC CM's inhibitory effect. Cysteine proteases degrade extracellular proteins and, in this case, could allow better penetration of antimicrobials into biofilms. This reasoning set up the final experiment where penicillin/streptomycin only disrupted mature MRSA biofilms when pretreated with MSC CM and not with oxacillin or penicillin/streptomycin itself (Marx et al., 2020).

Turning to canines, *in vivo* studies involving multidrug resistant (MDR) bacteria were recently conducted by Johnson et al. (2017). While the experiments were split between murine and canine models, the canine study is notable in that an observational pilot study was run evaluating poly I:C-activated allogeneic AT-MSCs in client-owned dogs with spontaneous MDR chronic wound infections. A series of three intravenous administrations at 2 week intervals of preactivated allogeneic canine AT-MSCs (2 × 10^6^/kg body weight) were given to 7 dogs. Antibiotic therapy continued throughout the trial and bacterial cultures were obtained from the wound sites starting prior to treatment and continuing every 2 weeks thereafter. After 8 weeks, 5 of the dogs had completely cleared infections of either methicillin resistant *S. pseudointermedius* (MRSP) or a combination of *P. aeruginosa* (PA) and *E. coli* (Table 2). The other 2 dogs did show clinical improvement with one of them eliminating 2 MDR species, but not MRSP. No adverse reactions to the treatments were noted. The authors argue that there are direct and indirect mechanisms at play in activated MSC suppression of wound infections. The findings of the murine experiments showed that MSCs secrete not only AMPs to interact directly with bacteria but also secrete factors that enhance bacterial clearance through activation of host innate immune cells as mentioned. Neutrophils co-cultured with activated MSCs were shown to phagocytose bacteria at higher levels. In addition, monocytes co-cultured with activated MSCs were shown to migrate to wound sites more and promote the M2 phenotype of macrophages in infected tissues (Johnson et al., 2017). The authors conclude that the synergism between MSCs (or MSC-secreted AMPs) and antibiotics could offer a solution to attenuate a variety of MDR bacterial infections without leading to further resistance. Another study examining the antimicrobial effects of canine MSCs is notable in that it introduces an underexamined area of MSC antimicrobial research: how MSCs may block communication among bacteria, otherwise known as quorum sensing (QS). QS is important to biofilm formation and differentiation, and when disrupted can lead to bacteria being more susceptible to antibiotics (Diggle et al., 2007). Using a QS reporter strain of *E. Coli* that bioluminesces when QS signaling is elicited, the authors were able to detect antibiofilm effect of canine BM-MSC CM. A previous study by the group that ran proteomic analysis on the CM allowed for the authors to posit specific components to explain the antimicrobial effects observed including the AMPs apolipoprotein B and D, amyloid-β peptide, cathepsin B, and protein S100-A4 (Humenik et al., 2019). While proteins and peptides picked from the known content of the CM could suggest plausible cause for the antimicrobial effects detected, it should be emphasized that it is merely speculative at this point as no further experiments were designed to test these candidates in any way.

**Table 2:** Patient data from 7 pet dogs with spontaneous, chronic infections with MDR bacteria treated with activated MSC.

**Dog**	**Infection site**	**Infection duration**	**Organism(s)**	**Bacteriologic response (8 wks)**	**Clinical Response (8 wks)**
1	Post-operative stifle infection	12 months	MRSP	Eliminated	Resolved
2	Post-operative stifle infection	6 mos	MRSP	Eliminated	Resolved
3	Draining tract stifle	4 mos	MRSP	Eliminated	Resolved
4	Soft tissue injury-paw	4 weeks	PA, EC	Eliminated	Resolved
5	Infected bone plate	3 mos	MRSP, EC, Crny, Kleb	Eliminated (except MRSP)	Improved
6	Cervical abscess from pacemaker lead	24 mos	MRSP- 2 strains	Unchanged	Improved
7	Deep pyoderma-paws	9 mos	MRSP	Eliminated	Resolved

*PA, Pseudomonas aeruginosa; EC, Escherichia coli; MRSP, methicillin resistant Staphylococcus pseudointermedius; Crny, Corynebacterium sp.; Kleb, Klebsiella sp. From Johnson et al. (2017). Licensed under CC BY 4.0. No changes were made. To view a copy of this license, visit http://creativecommons.org/licenses/by/4.0/*.

In bovine studies, two recent independent articles examining MSC therapies for mastitis in dairy cows indicate that MSCs may become a useful tool for production animals as well (Lange-Consiglio et al., 2019; Peralta et al., 2020). In the first study, four-fold concentrated CM reconstituted from lyophilized allogeneic non-primed bovine amniotic tissue-derived MSC CM were used for both *in vitro* and *in vivo* studies (Lange-Consiglio et al., 2019). The *in vivo* study enrolled 48 dairy cows with either acute or chronic mastitis and compared CM versus antibiotic treatment. Cows were treated intramammarily with CM or antibiotics twice daily on 3 consecutive days. No significant differences were found between the two treatment groups regarding somatic cell counts (SCC, a milk quality indicator) in milk samples, and there was no mastitis recurrence in any of the CM-treated animals compared to a 67 and 100% relapse rate in acute and chronic antibiotic-treated control cases, respectively. The *in vitro* study had *S. aureus*-inoculated bovine mammary epithelial cell cultures set up with 10% MSC CM added at the time of inoculation, 4 h later, or not at all. After 24 h, epithelial cell survival rates were 90% for cultures when supplemented by CM at time 0, 61% when supplemented at 4 h, and 0% when no CM was added (Lange-Consiglio et al., 2019). In the second study, *S. aureus* mastitis was experimentally induced in 15 Holstein Friesian cows who were then treated intramammarily twice within 10 days with either allogeneic non-primed bovine AT-MSCs (2.5 × 10^7^/dose), antibiotics, or ringer lactate (negative control; vehicle) (Peralta et al., 2020). As in the Lange-Consiglio et al. paper just discussed, no differences were seen in SCC. However, there were significantly fewer CFU in the MSC-treated cows compared to the negative control between days 6 and 10 (Peralta et al., 2020). One question not examined in this paper is why they opted for a cellular treatment when this research group is the very same that tested the different formulations (e.g., concentrated or activated) of bovine MSC CM against *S. aureus* discussed above (Cahuascanco et al., 2019).

## 3. Effects of Antimicrobials on MSC Function

Antimicrobials have not only been used in medicine to treat infections but also prophylactically in cell culture (Skubis et al., 2017). It has been observed that antimicrobials can have an effect on MSCs during culture and even in local tissue cells after therapeutic implementation. Thus, until more is known, the therapeutic properties of MSCs can be potentially affected by some antimicrobials (Skubis et al., 2017). While no study has yet examined conventional antimicrobials' effect on MSC antimicrobial function, it is still worth examining their influence on other functions.

Experimentally, different classes of antimicrobials did have a significant effect on MSCs from different sources (Table 3). Aminoglycosides, such as gentamycin are widely used in equine practice, including in regional limb perfusion (Rubio-Martínez and Cruz, 2006) and intraosseous applications (Parker et al., 2010). Potential toxic osteonecrosis secondary to intraosseous perfusion of gentamicin raised questions in terms of the potential toxic effects of this antibiotic on stem cells (Parker et al., 2010). In fact, osteogenesis and chondrogenesis of human BM-MSCs decreased significantly when cultured with gentamicin in a dose-dependent manner (Chang et al., 2006). This has been observed in osteoblasts with the use of other aminoglycosides as well, such as tobramycin (Rathbone et al., 2011) and amikacin but only when using higher concentrations (>2,000 μg/mL) (Rathbone et al., 2011). In equine BM-MSCs, on the other hand, gentamicin had no effect on cell viability *in vitro*, but it reduced total RNA levels at higher concentrations (500 μg/mL) (Parker et al., 2012). In this same study, fluoroquinolones such as enrofloxacin also caused significant reduction in BM-MSC viability and total RNA levels (Parker et al., 2012). Finally, in AT-MSCs, antibiotics such as amphotericin affected growth and differentiation of these cells within 24 h of culture (Skubis et al., 2017), which was also observed with chloramphenicol use (Turani et al., 2015).

**Table 3:** Effects of different antimicrobials in MSCs.

**#**	**Antibiotic class**	**Antibiotic**	**Species**	**Study type**	**Aim**	**Outcomes**	**References**
1	β-lactams	Penicillin	Horse	*In vitro*	To investigate the effects of commonly used antibiotics in equine practice on BM-MSCs viability and gene expression.	Dose-dependent effect. Increased mRNA expression of TNC and COL1A1 at 50 μg/mL. No effect observed in BM-MSCs viability, total RNA concentration or mRNA expression at higher concentrations (up to 500 μg/mL).	Parker et al., 2012
2	Cephalosporins	Ceftiofur	Horse	*In vitro*	As described in row 1.	Dose-dependent effect. Increased mRNA expression of TNC and reduced TGF-βR2 expression at 50 μg/mL. Reduced total RNA concentrations at 500 μg/mL.	Parker et al., 2012
3	Aminoglycosides	Gentamicin	Horse	*In vitro*	As described in row 1.	Dose-dependent effect. Reduced mRNA expression of BCI2 and COL1A2 at 50 μg/mL. Reduced total RNA concentrations at 500 μg/mL.	Parker et al., 2012
4		Amikacin	Horse	*In vitro*	As described in row 1.	Dose-dependent effect. Increased mRNA expression of matrix components and decreased BCI2 expression at 50 μg/mL. Reduced BM-MSC viability and total RNA concentration at 500 μg/mL.	Parker et al., 2012
5	Quinolones	Enrofloxacin	Horse	*In vitro*	As described in row 1.	Dose-dependent effect. Reduced BM-MSC viability and total RNA concentrations at 200 μg/mL and 500 μg/mL. Increase in mRNA COL1A2 expression at 50 μg/mL.	Parker et al., 2012
6	Tetracyclines	Doxycycline	Human	*In vitro*/*in vivo*	To test if doxycycline reduces MMP, enhances chondrogenesis of human BM-MSCs and improves cartilage repair in an osteochondral defect model in rats	Enhanced chondrogenesis of BM-MSCs *in vitro*/*in vivo*.	Lee et al., 2013a
7		Minocycline	Human	*In vitro*/*in vivo*	To evaluate the beneficial effects of BM-MSCs and minocycline in an autoimmune encephalomyelitis mice model.	Increased immunomodulatory effect when applied with BM-MSCs *in vivo*.	Hou et al., 2013
8	Polypeptide antibiotics	Bacitracin	Human	*In vitro*	To investigate whether bacitracin affects osteogenic differentiation of BM-MSCs and the molecular mechanisms involved.	Increased osteogenic differentiation of BM-MSCs.	Li et al., 2018

*AT-MSC, adipose tissue-derived mesenchymal stromal cell; BCI2, apoptosis regulator; BM-MSC, bone marrow-derived mesenchymal stromal cell; COL1A1, collagen type 1 α-1; COL1A2, collagen type 1 α-2, TGF-βR2, transforming growth factor β receptor 2; TNC, tenascin C*.

Although many antimicrobials have an inhibitory effect, other classes of antimicrobials can potentiate MSC differentiation (Lee et al., 2013a). Tetracyclines, such as doxycycline was shown to enhance chondrogenic differentiation of human BM-MSCs, which was further confirmed *in vivo* (Lee et al., 2013a). In addition, oxytetracycline was shown to promote cartilage differentiation in pre-chondrocyte cell lines, promoting chondrogenesis in a dose-dependent manner (Hojo et al., 2010). Polypeptide antibiotics such as bacitracin potentiated osteogenic differentiation of human BM-MSCs in a dose-dependent manner, increasing intracellular alkaline phosphatase (ALP), collagen and mineralization, and upregulating the level of osteogenic genes (Li et al., 2018). Amphotericin B (AmB) and AmB-Cu improved osteogenesis of AT-MSCs in the presence of osteogenic-induction factors, including dexamethasone, β-glycerophosphate and L-ascorbic acid compared to control and penicillin-streptomycin treated cells (Skubis et al., 2017).

Antimicrobials were also demonstrated to potentially induce immunomodulatory effects of MSCs. This was observed with the use of minocycline in human BM-MSCs (Hou et al., 2013). This effect was later confirmed in an *in vivo* study, where hydrogel loaded with minocycline enhanced the wound healing phenotype of human BM-MSCs in culture compared to hydrogel alone in a rat wound model (Guerra et al., 2017).

Other antimicrobials did not have a measurable effect on MSCs. Cephalosporins such as ceftiofur did not present noticeable effects until higher concentrations were used (at 500 μg/mL), which caused reduction in total RNA obtained in equine BM-MSCs, suggesting toxic effects. In the same study, different concentrations of penicillin overall did not present a significant effect in cells (Parker et al., 2012).

It is important to consider that the combination of penicillin-streptomycin is frequently used in cell culture to avoid contamination. This combination of antibiotics is used when cells are expanded and selected in preparation for experiments, including experiments that aim to investigate antimicrobial effects in MSCs (Parker et al., 2012). Although the assumption is that the effects of using such antibiotics could be considered negligible, penicillin-streptomycin has been demonstrated to cause significant change in gene expression, cell regulation, and growth rate (Cohen et al., 2006; Ryu et al., 2017). Such influence can interfere directly with how cells respond to different stimuli *in vitro* and consequently, experimental results (Ryu et al., 2017). It is possible that the use of penicillin-supplemented media could influence a cell population's response (or lack of response) to certain antimicrobials during the experiments reported here through selection bias.

### 3.1. Mechanisms of Antimicrobial Effects on MSC Function

Mesenchymal stem cell behavior and expression of healing properties is governed by signaling pathways such as mitogen-activated protein kinase (MAPK/ERK), transcriptional nuclear factor-kB (NF-kB), and c-Jun NH2-terminal kinase (JNK/SAPK) explained in detail elsewhere (Zhong et al., 2012; Choi et al., 2014; Lu et al., 2016). Such pathways are stimulated by pro-inflammatory cytokines, triggering the pro-healing phenotype in MSCs. Certain antibiotics have been shown to interfere with such pathways in MSCs (Guerra et al., 2017). One study demonstrated that the NF-kB pathway may be activated through the interaction of minocycline in MSCs, leading to the increase in IL-6 and VEGF observed (Guerra et al., 2017). The activation of the NF-kB pathway with the use of minocycline may occur due to the stimulation of TNF receptor family. Stimulation of TNF receptors leads to NF-kB cytoplasmic phosphorylation and IkB kinase ε (IKKε) nuclear translocation, which may be responsible for the minocycline-induced enhancement of VEGF and IL-6 production in MSCs (Guerra et al., 2017). The authors speculated that the increase in IL-6 could be related to increased internalization of *S. aureus* observed in MSCs treated with minocycline (Guerra et al., 2017). In addition, the activation of the NF-kB pathway is correlated with cleavage of complement protein C5 to C5a and C5b in MSCs. These complement proteins ultimately lead to the formation of a Membrane Attack Complex (MAC) that induces cell death in microorganisms (Manthey et al., 2009; Lappas et al., 2012). However, minocycline was observed to inhibit this specific effect (Guerra et al., 2017).

Regarding osteogenic differentiation, bacitracin has been shown to increase osteogenic differentiation in BM-MSCs (Li et al., 2018). Studies demonstrated that bacitracin activated the transcription of the bone morphogenetic protein-2 (BMP-2) gene, an essential gene in the BMP-2/SMAD signaling axis, leading to the phosphorylation of SMAD1/5/9, which was significantly increased in bacitracin-treated cells (Li et al., 2018). For chondrogenesis, cordycepin showed a regulatory effect in chondrogenic differentiation of MSCs. The increase in gene expression of chondrogenic genes was mediated by inhibition of Nrf2 and activation of BMP signaling (Cao et al., 2016).

However, as mentioned, some antibiotics may have a deleterious effect on cell differentiation. The mechanism of action of streptomycin is connected to its binding to the 30S subunit of the bacterial ribosome. It also possesses affinity to the ribosomes in eukaryotic cells. Streptomycin may act on eukaryotic cells through disruption of protein synthesis, affecting mitochondrial activity by binding to certain RNAs or by interfering with miRNAs' action (Li and Yue, 2019). This indicates that addition of streptomycin may change gene expression profiles in cells, thereby affecting the differentiation process.

Most antibiotics used either in clinical practice or cell culture seem to have some effect in MSCs. Such effects can be advantageous in certain cases when specific differentiation processes are targeted. With few exceptions, most antimicrobials seem to interfere with MSC proliferation, migration, and differentiation capacity. While these effects are usually concentration-dependent, clinicians and researchers should be conscious about the effects of antimicrobials and their implications in the clinical use of MSCs. More research into conventional antimicrobials' effect on MSC antimicrobial effect may be warranted, though the use of MSC CM may circumvent the problem altogether.

## 4. Conclusions

MSCs have shown promising efficacy in treating infections in proof-of-principle studies and small clinical case-studies. These observations warrant further study of MSCs' potential to replace or ameliorate current antimicrobial therapies. MSCs appear to exert their antimicrobial effects through a variety of mechanisms to induce microbial killing including AMP secretion, promotion of the host immune system, and direct phagocytosis. At this point, the number of studies examining MSCs as antimicrobials is quite limited in the veterinary space. However, piecing together the evidence from the few that exist suggests that MSCs can become a clinically important broad-spectrum antimicrobial effective in treating planktonic bacteria, biofilms, and MDR strains of bacteria alone or more likely in combination with conventional antimicrobials.

Much work remains to be done in order to determine the best approach to optimizing production conditions and MSC antimicrobial product formulation(s). Although preconditioning of MSCs with various stimulants has proven effective in inducing a more antimicrobial phenotype, comparison of these activation methods could be explored in more depth. While it is true that part of the overall effect comes from direct cell-cell contact, important for future clinical and commercial considerations is the confirmation that MSC secreted factors can be frozen, concentrated, and lyophilized for less expensive long-term storage without loss of antimicrobial activity. Fractionation of this cell-free product may also become an important step for improving potency or targeting certain species of bacteria.

There is also considerable justification to expand the focus to more veterinary species. Specific conditions that could be targeted include sepsis, pneumonia, urinary tract infections, and bloodstream infections. From a clinical perspective, veterinary patients merit continued efforts to improve their quality of care in and of themselves, and they may serve as powerful preclinical models of analogous human infections as in other areas of MSC research. From a “One Health” perspective, it is arguably even more important for human health that MSCs or cell-free preparations have the potential to curtail the use of antibiotics and decrease AMR in livestock where misuse may be most prevalent and pernicious. As this area of veterinary MSC research is only just emerging, there are a multitude of different research questions left to address.

## Author Contributions

KR, LG, and JW wrote sections of the manuscript. All authors contributed to manuscript revision, read, and approved the submitted version.

## Conflict of Interest

The authors declare that the research was conducted in the absence of any commercial or financial relationships that could be construed as a potential conflict of interest.

## Footnotes

^1^https://www.avma.org/resources-tools/avma-policies/joint-avma-fve-cvma-statement-responsible-and-judicious-use-antimicrobials (accessed September 3, 2020).

^2^https://www.canada.ca/en/public-health/services/antibiotic-antimicrobial-resistance/animals/actions/responsible-use-antimicrobials.html (accessed September 3, 2020).
